# Augmentation of haptic feedback for teleoperated robotic surgery

**DOI:** 10.1007/s11548-020-02118-x

**Published:** 2020-01-30

**Authors:** Philipp Schleer, Philipp Kaiser, Sergey Drobinsky, Klaus Radermacher

**Affiliations:** Chair of Medical Engineering, Helmholtz Institute for Biomedical Engineering, Pauwelsstraße 20, 52074 Aachen, Germany

**Keywords:** Surgical robotics, Synergistic systems, Shared control, Robotic manipulators, Human machine interaction, Haptics

## Abstract

**Purpose:**

A frequently mentioned lack of teleoperated surgical robots is the lack of haptic feedback. Haptics are not only able to mirror force information from the situs, but also to provide spatial guidance according to a surgical plan. However, superposition of the two haptic information can lead to overlapping and masking of the feedback and guidance forces. This study investigates different approaches toward a combination of both information and investigates effects on system usability.

**Methods:**

Preliminary studies are conducted to define parameters for two main experiments. The two main experiments constitute simulated surgical interventions where haptic guidance as well as haptic feedback provide information for the surgeon. The first main experiment considers drilling for pedicle screw placements, while the second main experiment refers to three-dimensional milling tasks such as during partial knee replacements or craniectomies. For both experiments, different guidance modes in combination with haptic feedback are evaluated regarding effectiveness (e.g., distance to target depth), efficiency and user satisfaction (e.g., detectability of discrepancies in case of technical guidance error).

**Results:**

Regarding pedicle screw placements a combination of a peripheral visual signal and a vibration constitutes a good compromise regarding distance to target depth and detectability of discrepancies. For milling tasks, trajectory guidance is able to improve efficiency and user satisfaction (e.g., perceived workload), while boundary constraints improve effectiveness. If, assistance cannot be offered in all degrees of freedom (e.g., craniectomies), a visual substitution of the haptic force feedback shows the best results, though participants prefer using haptic force feedback.

**Conclusion:**

Our results suggest that in case haptic feedback and haptic assistance are combined appropriately, benefits of both haptic modalities can be exploited. Thereby, capabilities of the human–machine system are improved compared to usage of exclusively one of the haptic information.

## Introduction

The concept of synergistic robotics has been proposed as an alternative to active surgical robots completely automating subsequences of a given surgical task [1]. Synergistic systems provide a simultaneous shared control where the surgical tool is cooperatively maneuvered by the surgeon and the robot. There are different realizations such as handheld or hands-on devices. However, teleoperated devices can potentially offer a wider and more versatile spectrum of cooperative functionalities [2]. They consist of a master console where the surgeon is inputting motion which is communicated to a slave robot which executes the commands. However, this physical decoupling of surgeon and situs commonly leads to loss of haptic feedback [3, 4]. A haptic information channel can not only be used to mirror forces and torques from the situs, which we will call *haptic feedback*, but also to provide spatial guidance according to a surgical plan, which we will call *haptic assistance* [2]. Research on haptic feedback indicates that information on tissue can be obtained, precision is increased and error rates as well as force peaks are reduced [5–8]. Furthermore, with haptic assistance defined volumes (e.g., bone) can be removed or sensitive tissues can be protected based on the concept of ‘virtual fixtures.’ Thereby, effectiveness and efficiency of task execution can be increased while reducing the workload for the surgeon [9–13]. However, when haptic feedback and haptic assistance is combined on the same haptic interface they can overlap and mask each other and make it impossible for the surgeon to distinguish between the two [14, 15]. Therefore, this study investigates how to augment haptic information for surgical tasks. Initially, preliminary studies were carried out to define parameters for the main experiments and subsequently two main experiments based on simulated surgical procedures were conducted. All experiments are performed using a Phantom OMNI (Sensable Technologies, Wilmington, MA, USA) in association with the real-time control software QUARC (Quanser, Markham, ON, Canada) and computer-based virtual test scenarios.

## Preliminary studies

The preliminary studies (PS1-3) were conducted with 10 human subjects (2 females, 8 males; age 21-35; one left handed). Every participant performed every study in every mode. However, the sequence in which modes were presented to each participant was randomized to compensate for learning effects. The study consisted of three parts which addressed stimulus sensation and reaction times (PS1), perception of vibrations (PS2) and comparison of various input modalities (PS3).

### PS1: stimulus sensation and reaction times

Haptic feedback and haptic assistance have been considered as two different haptic ‘channels.’ Therefore, the leading question of the first part of the preliminary study was:

Which stimulus or superposition of multiple stimuli should be used to inform the surgeon about changes in the respective other haptic channel?

**Fig. 1:**
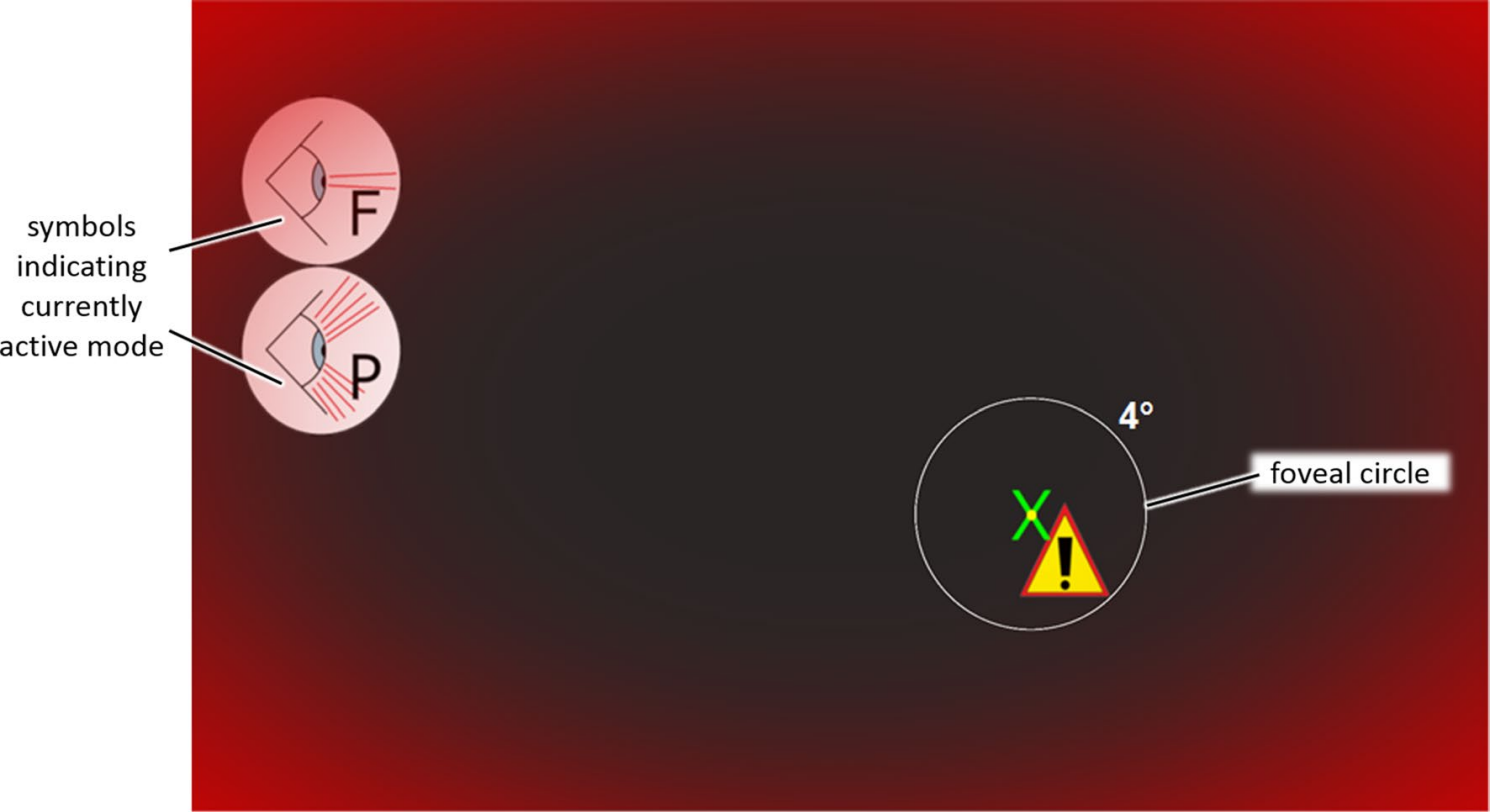
Graphical use interface (GUI) for the preliminary study of stimulus sensation and reaction times (4° circle of foveal field of view only inserted to support understanding)

Therefore, we designed a virtual test scenario to investigate which stimulus or combination of stimuli leads to faster reaction times and is perceived as pleasant by the user. To assure focus of the subjects onto a specific point on the screen, subjects had to follow a cross on the screen with the stylus of the haptic input device in their dominant hand (Fig. 1). Meanwhile, different stimuli were presented in a randomized order and participants were instructed to react by pressing the space bar with their non-dominant hand as fast as possible. Furthermore, participants were asked to answer a questionnaire and state how pleasant as well as how disturbing they perceived the signals on a five step Likert scale. The different stimuli were designed as follows:

- Tactile—vibration of 250 Hz according to the literature [16], heuristically determined intensity of 0.12 N
- Auditory—superposition of sinuses with 3000 Hz and 250 Hz based on recommendations of the DIN EN ISO 9241-171 displayed via circumaural headphones
- Peripheral (Visual)—red semi-transparent frame at the edges of the screen
- Foveal (Visual)—caution sign next to the cross to be tracked,

The foveal and the peripheral signal were separated according to a 4° cone [17] and the estimated distance of the subjects from the screen (Fig. 1). The combinations of signals (modes) and related acronyms for each mode are found in Table 1. As the focus of the study is primarily haptics, the individual signals were tested as well as all combinations including a haptic stimulus (vibration).

**Table 1:** Signals and combinations for the preliminary study of stimulus sensation and reaction times

Tactile (vibration)				X	X	X	X	X	X	X	X
Auditory			X					X	X	X	X
Peripheral		X				X	X			X	X
Foveal	X				X		X		X		X

Modes	F	P	A	V	VF	VP	VFP	VA	VFA	VPA	All

*F* foveal, *P* peripheral, *A* auditory, *V* vibration

**Fig. 2:**
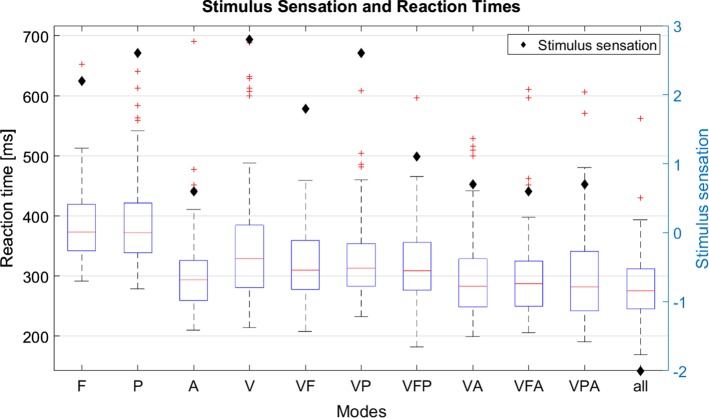
Stimulus sensation (pleasantness—disturbance; maximum standard error ≈ 0.9) and reaction time in ms for the different signals and combinations (*F* foveal, *P* peripheral, *A* auditory, *V* vibration)

Concise results of the experiment are illustrated in Fig. 2. Outliers of more than 700 ms were excluded from the analyses which resulted in an exclusion of 12 signals out of the 1100 measurements (10 measurements × 10 participants × 11 modes). Tendencies of the results indicate that a superposition of multiple signals leads to faster reaction times which is in accordance with the literature [18, 19]. Furthermore, acoustic signals (A) or combinations involving acoustic signals (VA, VFA, VPA, all) are perceived the fastest, however, have a strong negative impact on signal sensation (questionnaire: disturbance score subtracted from pleasantness score). Furthermore, peripheral signals were rated slightly better than foveal signals and the combination of more than two signals simultaneously was perceived comparably negative. The combination of peripheral and tactile signals constitutes a good compromise between reaction time and stimulus sensation and will therefore be used for the first main experiments.

### PS2: perception of vibrations

The second preliminary study addresses the perception of vibrations with respect to the contact force between haptic interface and human operator. The force applied to a surface by the users finger influences the perception of vibrations [19, 20]. Therefore, we investigated which force amplitude should be displayed with respect to our system characteristics in dependence of the applied force. Results indicate that perceptibility of vibrations tends to increase as forces are applied in direction of the test person which is in accordance with the literature [19, 20]. However, as the effect was too small, it has been neglected for the main experiment. The values served for the design of vibratory signals for the first main experiment and were set to 0.075 N and 0.15 N for minimal vibration intensity and clearly noticeable vibration, respectively.

### PS3: input modalities

The literature provides information with respect to different input modalities such as ergonomic design of foot switches [21] and reaction times with respect to push buttons [19] and haptic devices [22]. However, data were incomplete for a final comparison of reaction times and recommendations for use for the four input modalities:

- Space bar on keyboard
- Foot switch with three pedals (Steute, Loehne, Germany)
- Button on the haptic device
- Retraction of the stylus (inspired by [22])

PS3 was based on the setup of PS1; however, here only the tactile stimulus was presented throughout the whole experiment. A symbol on the GUI indicated the randomized input modality to confirm the stimulus. Thereby, influences with respect to the spatial proximity of the sensory neurons (i.e., stimulus perception) and the motor neurons (e.g., finger, foot), which were assumed in [22], could be investigated. Ten consecutive measurements were taken for each input modality and participants answered a questionnaire rating the following statements on a five level Likert scale:

- The trigger inferred with the execution of the tracking task.
- Keeping the trigger in constant readiness to react is pleasant.
- Triggering was pleasant for me.
- To give a planned, infrequent input (about 1 × per minute), the trigger is pleasant.
- Holding down the trigger continuously is pleasant. (Was not asked for retraction of the stylus.)

**Fig. 3:**
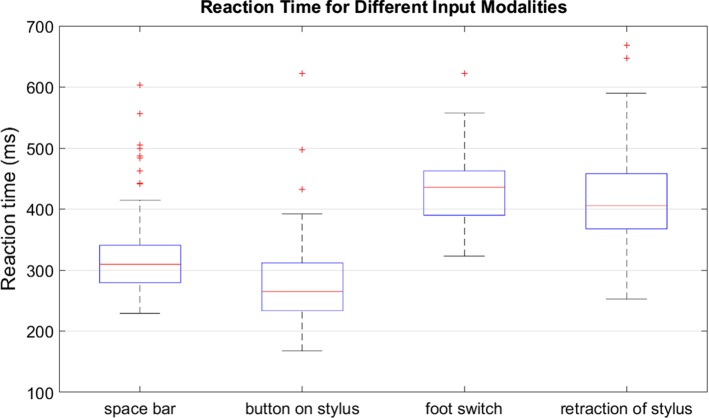
Reaction time for different input modalities in ms

Results illustrated in Fig. 3 indicate that shorter reaction times are achieved with the button on the stylus, followed by the space bar and finally the foot switch which supports assumptions in the literature [22]. Measurements of the foot switch, however, could be influenced by the latency due to the wireless connection. Latencies with respect to the haptic device and the space bar can be assumed to be similar. Deviations with regard to the tracking task indicate that there is no difference between input modalities except for the retraction of the stylus, where deviations are higher.

**Fig. 4:**
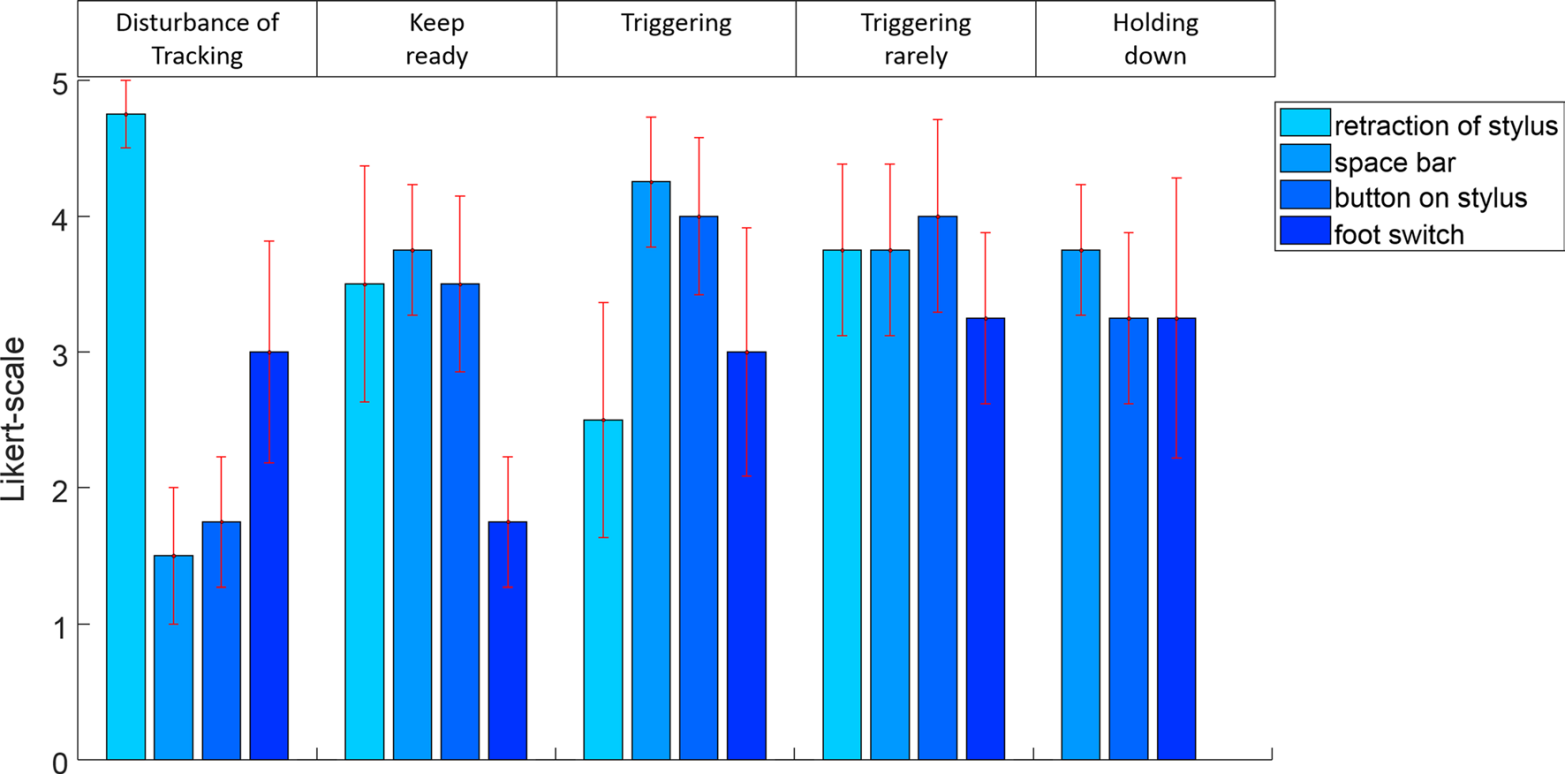
Results of the questionnaire regarding the different input modalities (error bars indicate the standard error)

Questionnaire results show least influence on the tracking task by the button on the stylus and the space bar followed by the foot switch (Fig. 4). Keeping ready to react is comparably less comfortable with the foot switch and triggering in general is most pleasant with the space bar and the button on the stylus. For planned infrequent inputs as well as holding down the trigger the button on the stylus, the spacebar and the foot switch are appropriate.

In summary, for infrequent inputs and holding down the trigger, the foot switch represents an alternative to the button on the stylus and the space bar, but reaction time is worse. When reaction time is important, the button on the stylus should be preferred over the space bar if there are haptic signals involved. Retraction of the stylus was not considered for the main experiments due to the negative effects on the tracking task. Findings of PS3 were used to select the input modalities for the versatile mode of the second main experiment.

## Main experiments

To evaluate different ways to combine haptic feedback from the situs with haptic assistances, two main experiments were designed which are based on surgical procedures where superposition of haptic feedback and haptic assistance could be beneficial. The first experiment constitutes a simulated pedicle screw placement, while the second experiment addresses milling tasks such as performed during partial knee replacements or craniectomies.

### Pedicle screw placement

For the placement of pedicle screws, the exact positioning and orientation are of major importance [23, 24]. However, the navigation system’s accuracy decreases with insertion depth due to reference markers being placed externally on the spine [25]. Therefore, the drilling force progression, which is independent of the referencing of the navigation system, can be an important complementary information. An outer cortical bone layer and cancellous bone inside leads to a distinct force profile during drilling [26].

An experiment was designed to evaluate the superposition of haptic feedback and haptic assistance during depth control of a simulated drilling task. Participants were advised to execute several drillings orthogonal to the visual display plane. They were haptically guided on a linear trajectory while a simulated force profile based on [26] was displayed along the drilling trajectory. The force profile was scaled down to a maximum force of 2.5 N due to limitations of the haptic device used. To reach the target depth at the transition between cancellous bone and ventral cortical bone [26] as accurately as possible, different combinations (Table 2) of the following augmentations were presented:

**Table 2:** Signals and combinations for the main experiment regarding pedicle screw placement

*Assistances*							
Visual (*V*)—scale			X		X		X
Tactile (*T*)—scale				X			
Binary (*B*)—tactile (vibration) + peripheral		X	X	X			
Wall (*W*)—kinesthetic (force) + peripheral						X	X
*Haptic feedback (F)*	X	X	X	X	X	X	X
Modes	F	FB	FVB	FTB	FV	FW	FVW

*F* haptic feedback, *B* binary (vibration + peripheral), *V* visual (scale), *T* tactile (scale), *W* wall (kinesthetic + peripheral)

**Fig. 5:**
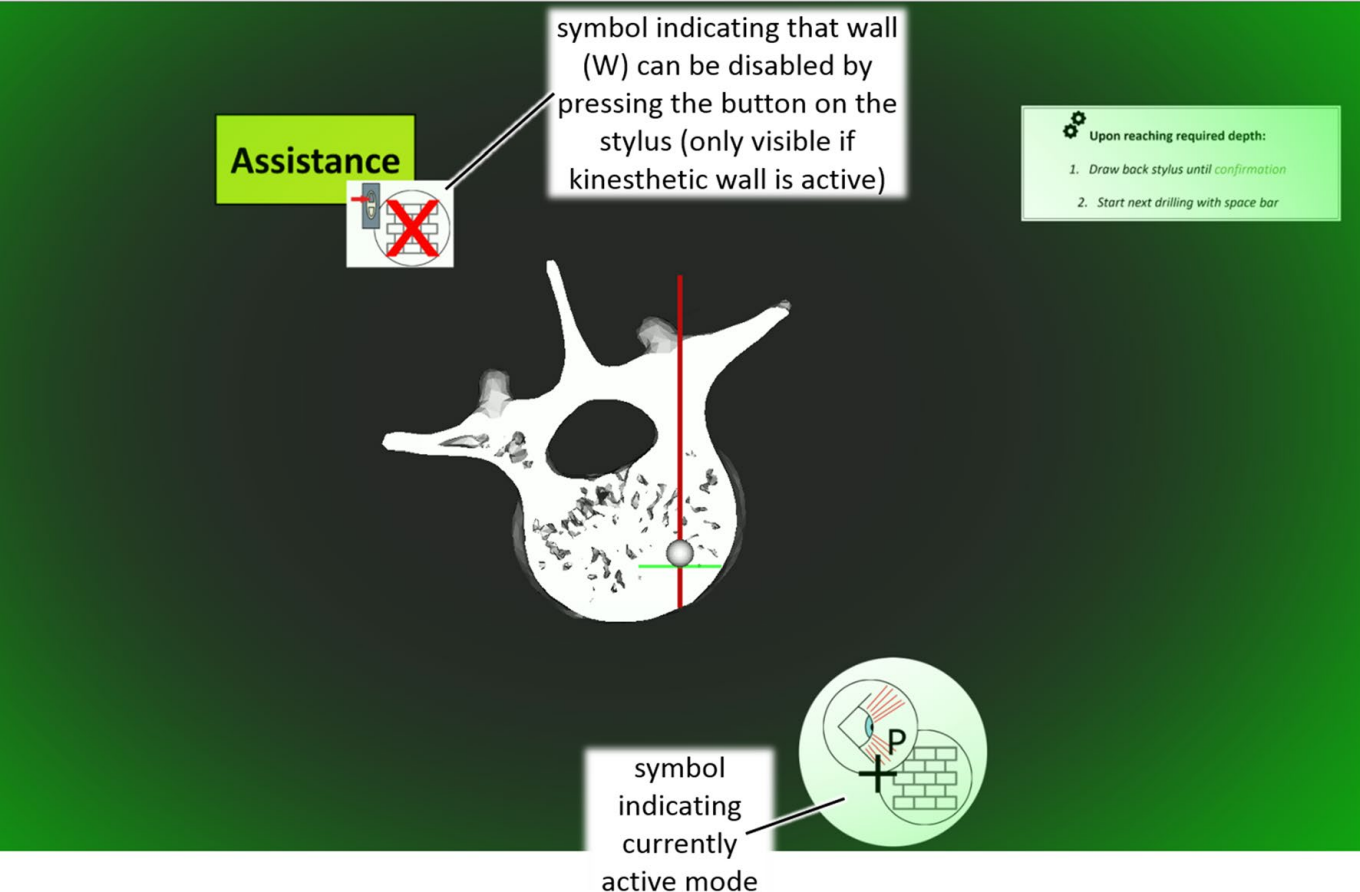
Graphical user interface for the first main experiment regarding pedicle screw placements

- **Visual** (**V**) representation of the current depth indicated by a gray sphere
- **Tactile** scale (**T**) representing the current depth by an increasing rate of vibrations until a constant vibration at the target depth
- **Binary** tactile and peripheral signal (**B**) at the target depth based on the results of the preliminary study (PS1)
- Kinesthetic **wall** (**W**) (stiffness 1.05 N/mm) and peripheral signal at target depth. The wall could be disabled by pressing the button on the stylus which is also indicated on the GUI (Fig. 5)

The selection of the combinations in Table 2 was chosen for the following reasons:

- *Haptic Feedback (F)*—serves as a reference for an execution without assistance and solely haptic feedback
- *Haptic Feedback*** + ***Binary (FB)*—additional binary feedback (vibration + peripheral) at the target depth but without a visual hint about the progress during execution
- *Haptic Feedback*** + ***Visual*** + ***Binary (FVB)*—extends FB with a visual indication of the current depth (gray sphere) to evaluate its influence
- *Haptic Feedback*** + ***Tactile*** + ***Binary (FTB)*—replaces the visual indication of the current depth of FVB by a tactile scale
- *Haptic Feedback*** + ***Visual (FV)*—serves as a reference for the other modes with a visual scale but without additional assistance (e.g., if optical tracking is used during a surgery)
- *Haptic Feedback*** + ***Wall (FW)*—comparable with FB but differs in that the vibration was replaced by a kinesthetic wall and represents a mode in which haptic feedback and haptic assistance are superpositioned in the same degree of freedom
- *Haptic Feedback*** + ***Visual*** + ***Wall (FVW)*—extends FW with a visual indication of the current depth (gray sphere)

Thereby, the visual representation (*V*) is intentionally omitted in some combinations as the visual channel is dominant such that users trust it until they have sufficiently strong evidence to overrule it [27]. This is particularly interesting as during some trials an inaccuracy in the guidance information was artificially generated such that all guidances/augmentations (subjected to registration errors) and the force profile along the trajectory (independent of registration errors) contradicted each other.

Before the experiment, every participant was briefed in a standardized way about bone anatomy and the related force profile, how the target depth is defined and how it is reflected in the force profile. Participants were informed about possible discrepancies between haptic feedback and augmentations and that in case of conflict the haptic feedback is more reliable as it is independent of registration accuracy. Consecutively, participants underwent a trial to familiarize with the different augmentation modes and the investigator ensured that the haptic wall (*W*) was deactivated at least once. Afterward, five consecutive drillings were performed for each mode wherein an early and a delayed presentation of the guidance information in comparison with the target depth were presented in a randomized order. Following each execution, participants were asked if they detected a discrepancy between the haptic feedback and the guidance information. The sequence of the different modes was randomized for each participant. Dependent variables were chosen based on DIN EN 60601-1-6 and constituted of:

- Effectiveness—distance to target depth
- User satisfaction—situational awareness (detection of discrepancies)

Efficiency was not evaluated as users were told to take about 10 to 20 s per drilling to simulate an execution close to reality.

### Milling (partial knee replacement, craniectomy)

In partial knee replacements, malalignments can lead to loosening of prostheses components and excessive wear [28]. Therefore, an exact preparation of the implant cavity is crucial and common registration accuracy standards are sufficient for assistance during milling. In case of craniectomies, bone is removed by milling if an adhesion of the dura mater and the skull bone is suspected [29]. However, due to inaccuracies in image acquisition (CT-Scan resolution ~ 0.45 mm [30]) and optical tracking (accuracy ~ 0.5 mm [31, 32]) security offsets of at least 1 mm from the inner bone surface need to be planned preoperatively [30]. Due to this uncertainty, no assistances based on planning data can be offered within the safety offset. Here, the force profile during milling [33] could serve as an information source for the surgeon, in order to preserve the dura and be able to modify the outer contour of the craniectomy according to intraoperative requirements [29].

**Fig. 6:**
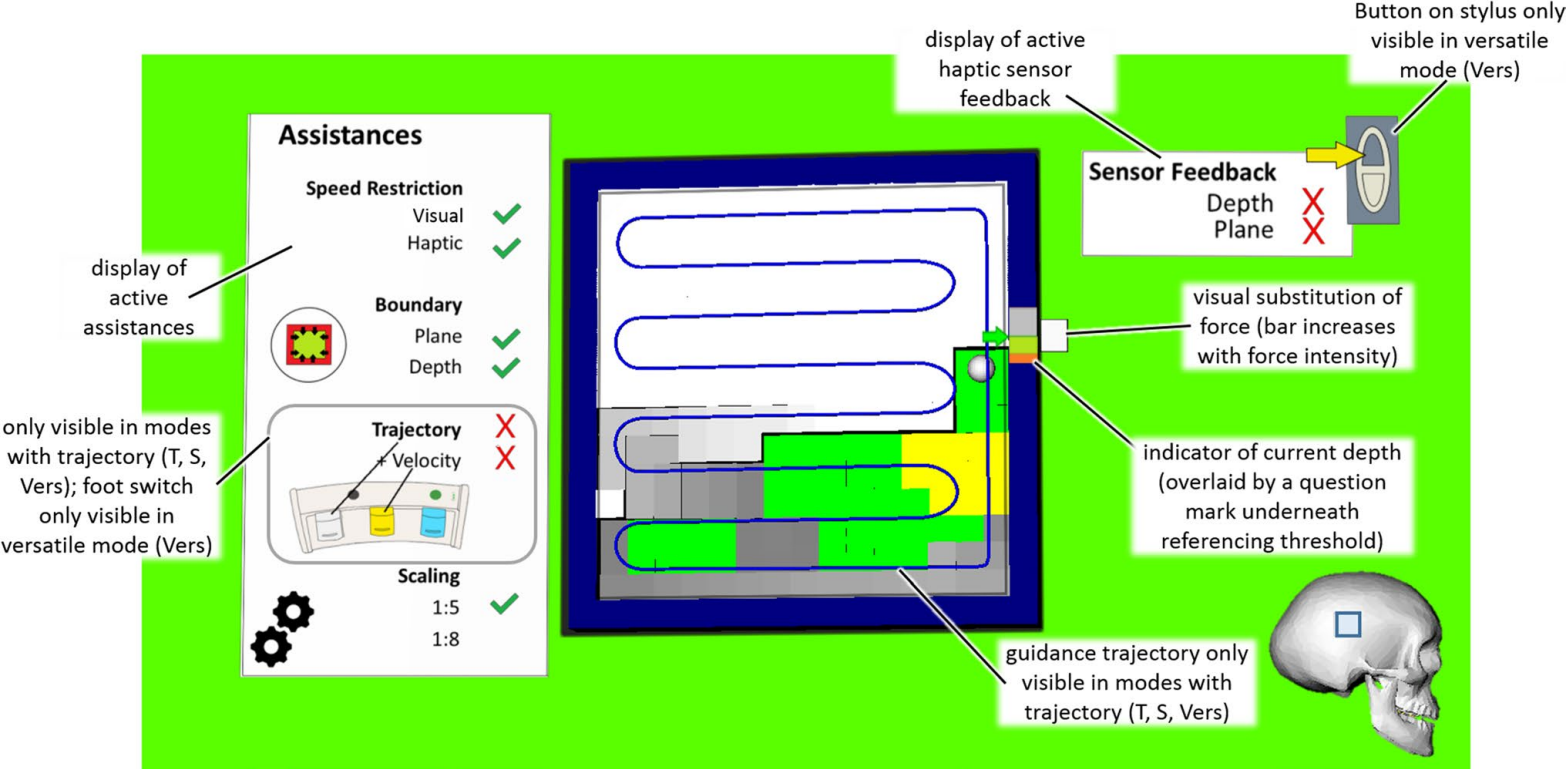
GUI for the second main experiment regarding milling; depth of the individual voxels is color-coded; background indicates active forces in depth direction (haptic guidance ≙ green, haptic feedback ≙ yellow, no force ≙ white)

Therefore, the experiment is divided into two phases with the first phase (P1) targeting milling tasks which can be assisted in the entire planned volume (e.g., partial knee replacements). The second phase (P2) targets interventions (e.g., craniectomies) where due to limitations of the acquired data (i.e., image acquisition resolution and optical tracking), assistances cannot be offered in all degrees of freedom (DOF). Below a certain security offset (approximately 1 mm [30]), haptic feedback in the depth direction has to be relied upon, while haptic guidance is offered in the remaining DOFs, enabling a synergistic path control [12]. A simulated haptic feedback was implemented based on the force profile in [33]. The GUI and descriptions of the visual elements are illustrated in Fig. 6 and constituted of two displays one for the active assistances and one for the active haptic feedback. The trajectory itself as well as the indicator on the active assistance panel was only visible in modes with a trajectory (*T*, *S*, Vers). A visual substitution of the force applied in the depth direction was visible in every mode as a white bar next to the cursor which increased in size depending on the simulated haptic feedback. Additionally, the current depth was displayed by a color-coded bar next to the cursor which was overlaid by a question mark as soon as the cursor is moved underneath the referencing threshold in phase 2 to indicate that the display is no longer reliable. The depth of the individual voxels was color-coded and the background indicated which forces are active in the depth direction (haptic guidance ≙ green, haptic feedback ≙ yellow, no force ≙ white).

Haptic and visual assistance constituted of:

- **Speed restriction**—warning indicating that the current speed is higher than 30 mm/s, either by a red transparent frame at the edges of the screen with increasing intensity (visual) or by an increasing force opposite to the direction of movement (haptic)
- **Boundary**/**Constraint** (haptic)
   Plane—increasing force starting 5 mm before the edges of the cavity with a stiffness of 0.5 N/mm in the main visual plane
   Depth—increasing force with a stiffness of 0.3 N/mm in the depth direction at the target depth (Phase 1) or the referencing threshold (Phase 2)
- **Trajectory** (haptic)—guidance based on the deviation from an optimal milling path with a stiffness between 0.05 and 0.09 N/mm depending on scaling
   + Velocity (haptic)—guidance along the path to maintain a constant velocity of 20 mm/s
- **Scaling** (depth direction)—scaling of 1:5 in the depth direction was used in every mode except for the scaled mode where it was increased to 1:8.3.

Combinations of assistances for the different modes and phases of the experiment are gathered in Table 3 and were chosen for the following reasons:

**Table 3:**
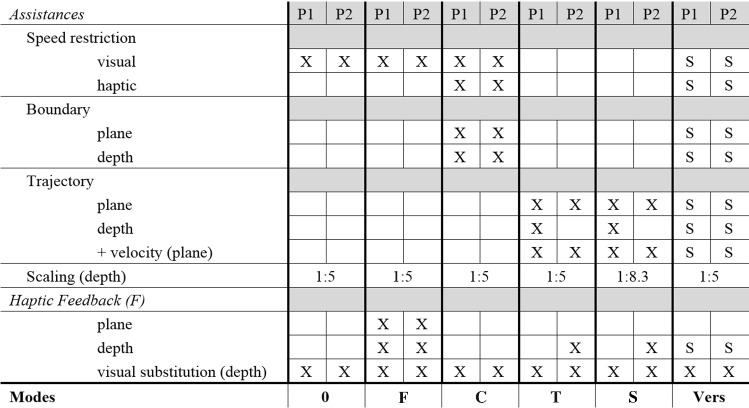
Signals and combinations for the main experiment regarding milling

0 no haptic feedback, *F* haptic feedback, *C* constraint, *T* trajectory, *S* scaled, *Vers* versatile (users can turn several modalities on/off indicated by a *S* for selectable)

- *No Haptic Feedback (0)*—serves as a reference without haptic feedback and solely visual substitutions.
- *Haptic Feedback* (*F*)—extends mode 0 with haptic feedback.
- *Constraint* (*C*)—constitutes a haptic assistance mode where the user is constrained by virtual boundaries around the cavity to be prepared in both phases.
- *Trajectory* (*T*)—haptic assistance mode where a specific trajectory (i.e., milling path) is predefined. For P2, assistance in the depth direction is deactivated and replaced by haptic feedback.
- *Scaled* (*S*)—comparable with mode *T* but differs in that scaling in the depth direction is increased.
- *Versatile* (*Vers*)—participants could turn assistances on or off depending on their preference also displayed on the user interface (Fig. 6) and based on the findings of PS3. By pressing the white foot pedal once, they were guided toward the closest point on the trajectory and afterward along the trajectory. Pressing the white foot pedal again turned off the trajectory guidance. Pressing and holding down the yellow foot pedal activated/deactivated the velocity guidance if the trajectory was activated. Holding down the button on the stylus activated haptic feedback in the depth direction and deactivated any guidance forces in the respective direction. The default mode at the beginning of the execution corresponds to the settings of the *C* mode.

**Fig. 7:**
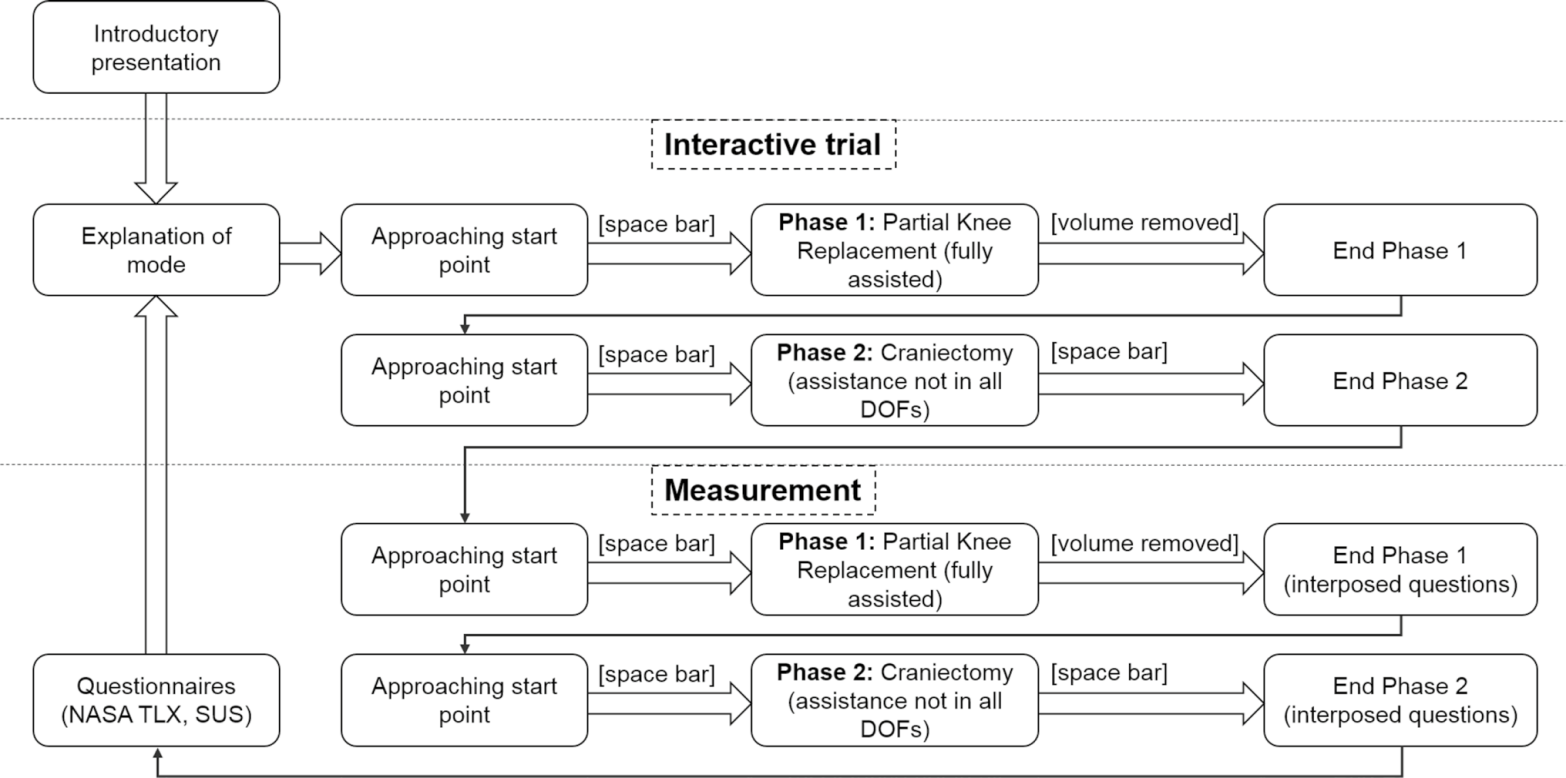
Process diagram of the second main experiment

The process of the second experiment is visualized in Fig. 7. The introductory presentation included information about the two phases of the experiment, the meaning of the force profile and why no assistance can be offered underneath the safety offset. Participants were advised to move as precisely and time efficient as possible. Modes without trajectory guidance (0, *F*, *C*) were tested first and afterward the remaining three modes (*T*, *S*, Vers) were tested in a randomized order. Before every experiment execution, an interactive trial was presented to participants to familiarize themselves with the current mode before the measurement was recorded. After each phase, the following interposed questions were answered on a scale from 0 to 100%:

- How big is your influence on task execution?
- How strongly do you feel restricted in your free action?

After each mode, participants filled out the NASA-TLX rating scales [34] and the System Usability Scale (SUS) questionnaire [35] and following the whole second main experiment the NASA-TLX source of workload [34].

Dependent variables were chosen based on DIN EN 60601-1-6:

- Effectiveness—mean deviation from goal depth
- Efficiency–duration
- User Satisfaction—based on NASA-TLX and SUS

## Results

Thirteen participants (4 females, 9 males; age 21–28; two left handed) took part in the experiments of which none had prior experience with the system. Not every participant took part in both experiments which resulted in 10 measurements per experiment. Every participant performed an experiment in every mode. However, the sequence in which modes were presented to each participant was randomized to compensate for learning effects. Differences between assistance modes were evaluated using analysis of variance (ANOVA) with a post hoc test using the Tukey–Kramer method with an alpha level of 0.05.

### Pedicle screw placement

The dataset of the first experiment consists of 50 measurements for the reference mode (*F*). Every other mode consists of 30 measurements without discrepancies and additionally 10 measurements each for early and delayed presentation of the assistance (± 7 mm to target depth). Measurements where drillings were aborted although neither force feedback nor assistances gave any reason to do so were excluded

**Fig. 8:**
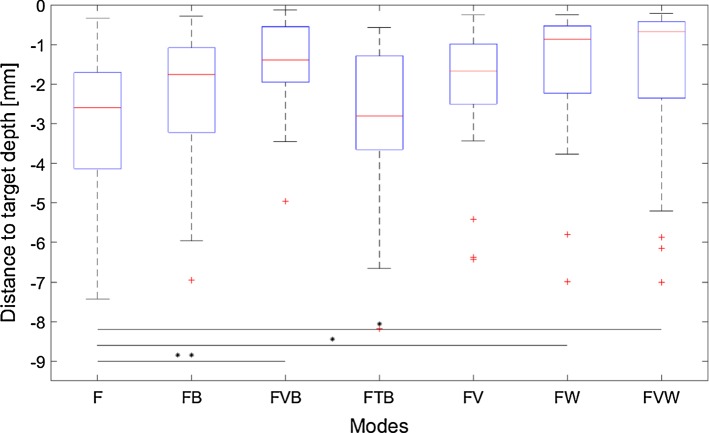
Distance to target depth in mm for drillings without discrepancies (*p < 0.05; **p < 0.01; ***p < 0.001) (*F* haptic feedback, *B* binary (vibration + peripheral), *V* visual (scale), *T* tactile (scale), *W* wall (kinesthetic + peripheral))

Results regarding effectiveness in Fig. 8 indicate a statistically significant difference in target depth deviation (*F*(6, 214) = 3.78; *p* = 0.0014).

Questionnaire results in Table 4 are classified as hit, correct rejection, miss and false alarm according to signal detection theory. Additionally, misses are further differentiated depending if they happened during early or delayed presentation of the assistance. During the early case, the assistance indicated that the goal depth was reached, even if participants should have moved further to reach the real target depth. For the delayed case, participants felt an increasing force due to the haptic feedback of the real target depth before the assistance indicated the goal depth (Note: They should have stopped the movement before the assistance indicated to do so.)

**Table 4:** Detection of discrepancies between haptic feedback and assistance based on a question following each execution

	FB	FVB	FTB	FV	FW	FVW
Correct rejection	27	24	26	27	25	29
False alarm	3	6	4	3	5	1
Hit	17	17	15	18	12	13
Mass	3	3	5	2	8	7
During early presentation	3	2	3	1	6	6
During delayed presentation	0	1	2	1	2	1

*F* haptic feedback, *B* binary (vibration + peripheral), *V* visual (scale), *T* tactile (scale), *W* wall (kinesthetic + peripheral)

### Milling (partial knee replacement, craniectomy)

Results of the second experiment consist of one measurement per participant, which results in 10 measurements for each mode.

**Fig. 9:**
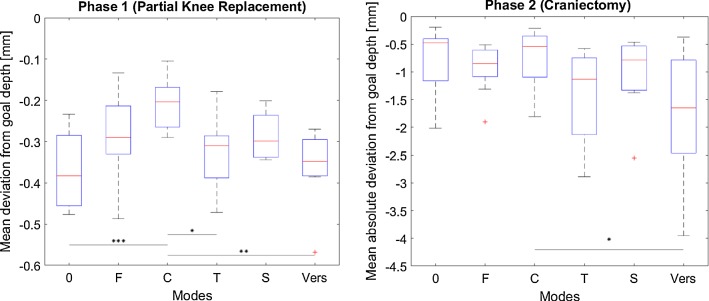
Mean (absolute) deviation from goal depth in mm for milling (*p < 0.05; **p < 0.01; ***p < 0.001) (0 no haptic feedback, *F* haptic feedback, *C* constraint, *T* trajectory, *S* scaled, *Vers* versatile)

Figure 9 gathers the results regarding effectiveness for both phases (P1, P2). Distances refer to the virtual environment coordinates as different scaling factors were used. Statistically significant differences were found for P1 (*F*(5, 54) = 5.45; *p* < 0.001) and P2 (*F*(5, 54) = 2.95; *p* = 0.02).

**Fig. 10:**
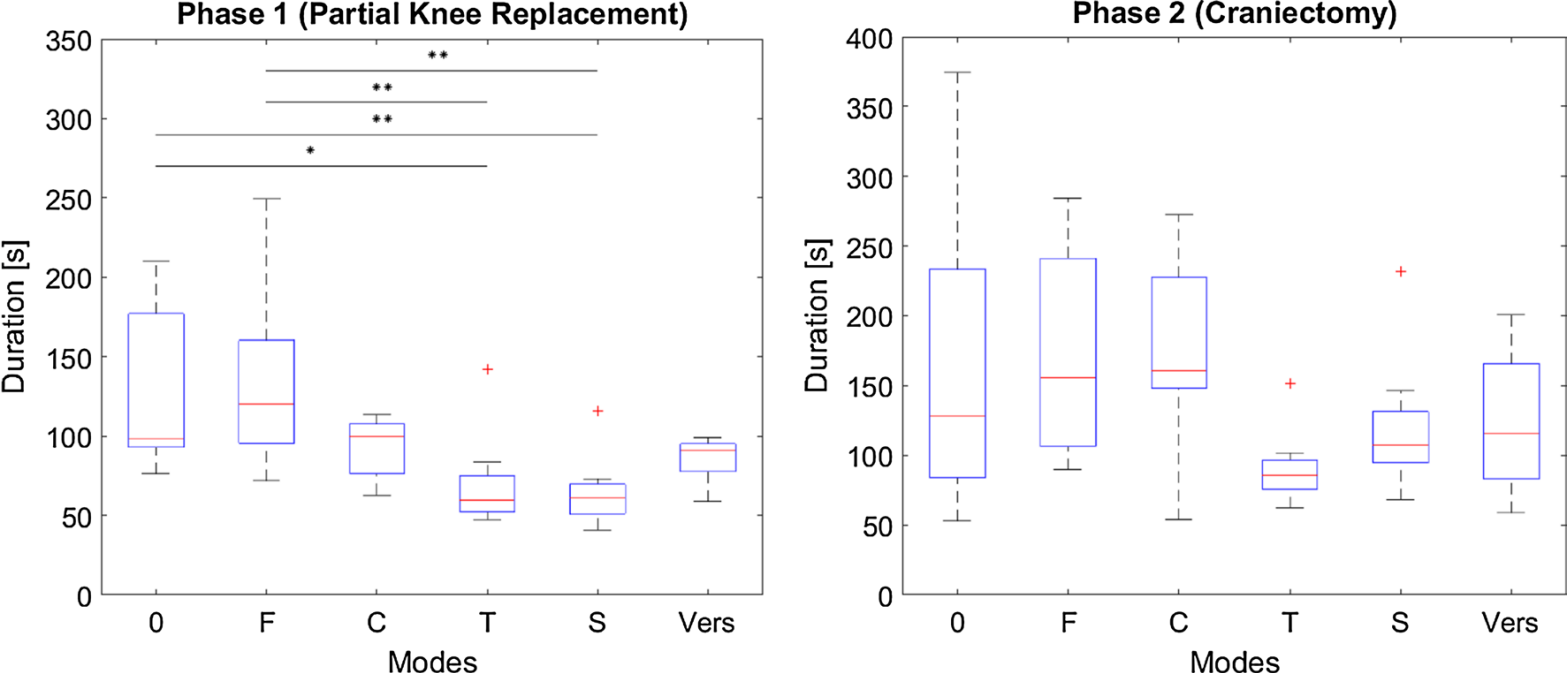
Duration in s of milling (*p < 0.05; **p < 0.01; ***p < 0.001) (0 no haptic feedback, *F* haptic feedback, *C* constraint, *T* trajectory, *S* scaled, *Vers* versatile)

Results regarding efficiency are illustrated in Fig. 10. For P1, 4 of the 30 measurements of the modes *T*, *S* and *V* were excluded as participants moved against the direction of the velocity guidance had difficulties navigating through the corners or did not realize that the experiment has already started. For P2, one measurement was excluded as the participant moved against the direction of the velocity guidance. A statistically significant difference could be found for P1 (*F*(5, 50) = 6.03; *p* = 0.0002) and P2 (*F*(5, 53) = 2.52; *p* = 0.041).

**Fig. 11:**
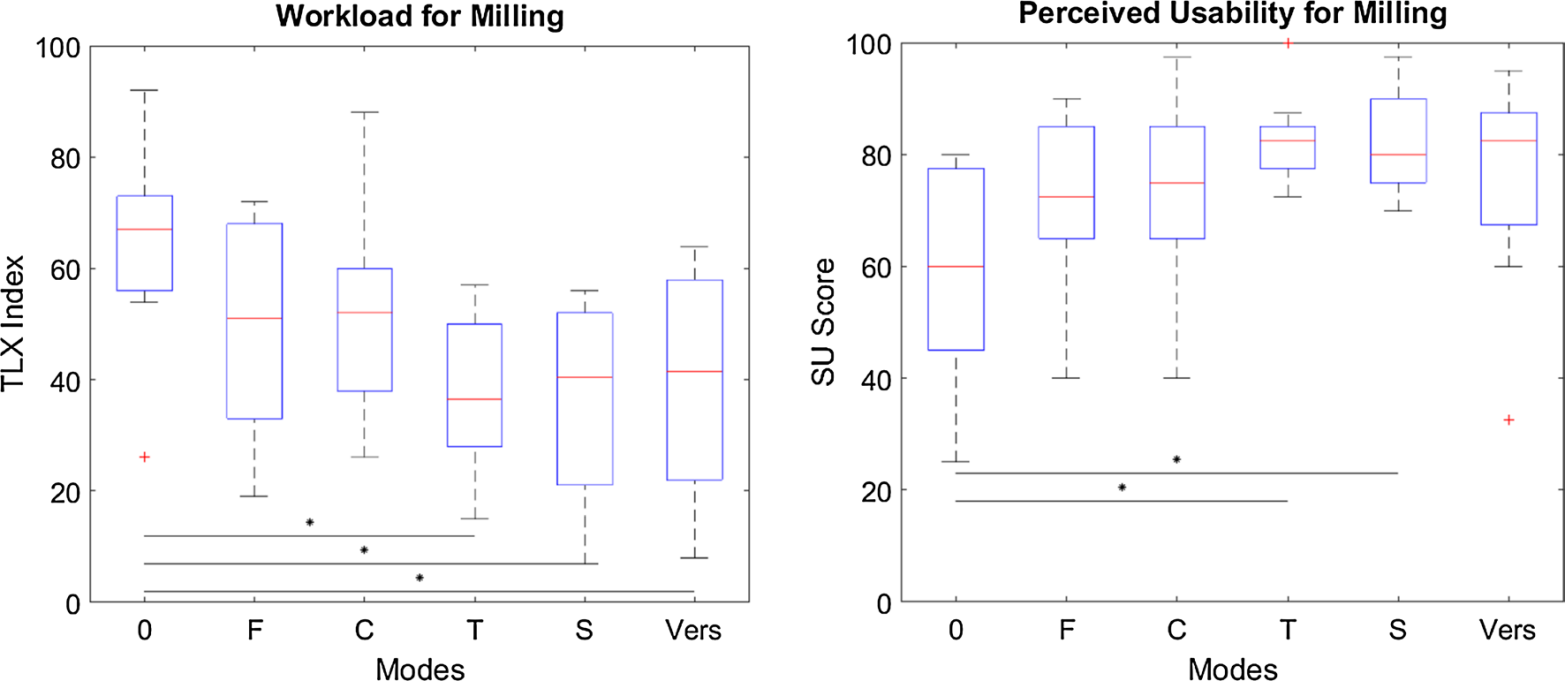
User satisfaction for milling (*p < 0.05; **p < 0.01; ***p < 0.001) (0 no haptic feedback, *F* haptic feedback, *C* constraint, *T* trajectory, *S* scaled, *Vers* versatile)

A statistically significant difference is observed for the results of the NASA-TLX (*F*(5, 54) = 3.58; *p* = 0.0072) and SUS (*F*(5, 54) = 3.26; *p* = 0.0122) questionnaire illustrated in Fig. 11. Results of the interposed questions on perceived influence of participants on task execution and perceived restriction by assistance are listed in Table 5. Answers of one participant were excluded as he misinterpreted the interposed questions.

**Table 5:** Perceived influence of participants on task execution and perceived restriction by assistance

		Modes					
		0	F	C	T	S	Vers
*Perceived influence on task execution (in* %*)*							
Phase 1 (partial knee replacement)							
Mean		89.4	87.8	83.9	58.3	59.4	76.7
SD		15.5	12.8	14.7	16.6	17.4	19.8
Phase 2 (craniectomy)							
Mean		87.8	86.7	78.9	66.1	53.9	81.7
SD		16.4	13.2	17.5	19.3	22.3	20.3
*Perceived restriction by assistance (in* %*)*							
Phase 1 (partial knee replacement)							
Mean		5.5	8.0	9.7	37.7	43.0	18.0
SD		6.9	7.9	9.5	19.3	21.2	21.1
Phase 2 (craniectomy)							
Mean		5.0	9.0	9.0	32.7	43	16.5
SD		8.2	7.7	9.7	21.9	22.8	18.6

0 no haptic feedback, *F* haptic feedback, *C* constraint, *T* trajectory, *S* scaled, *Vers* versatile

## Discussion and conclusion

This paper analyzes different ways to combine and augment *haptic feedback* from the situs and several *haptic assistances* for planning guided teleoperated robotic surgery. Results of the preliminary study were in accordance with findings in the literature and successfully served for the design of the main experiments. Two main experiments were designed based on surgical procedures, namely pedicle screw placements and milling tasks such as partial knee replacements or craniectomies.

### Pedicle screw placement

Results regarding effectiveness of the different augmentations indicate statistically significant improvements in target depth deviation by the augmentation of haptic feedback in case of no simulated discrepancies between assistance and force feedback information. Three modes significantly improved drilling accuracy: both modes which incorporate a kinesthetic wall (FW, FVW); a visual scale in combination with a binary confirmation (vibration + peripheral signal) at the target depth (FVB). No statistically significant improvements could be observed for the visual scale or the binary confirmation alone confirming the literature that the combination of several feedback modalities leads to further improvements (e.g., reaction time) [18, 19]. However, with respect to the detection of discrepancies between *haptic feedback* and *haptic assistance* the two modes containing a kinesthetic wall show the highest amount of misses in comparison with any other mode. In case of an early presentation of the assistance, 12 out of the 20 discrepancies were not detected, supporting the literature that feedback and assistance forces should not be superposed on the same DOF [14, 15]. In case of a delayed assistance, no statistically significant deterioration with regard to deviation from the target depth is observed. Hence, the expectation of the participants to be supported by an assistance does not significantly degrade performance in case of a delayed assistance signal. In conclusion, mode FVB constitutes a good compromise between increasing effectiveness and detection of discrepancies between assistance and force feedback information.

### Milling (partial knee replacement, craniectomy)

**Fig. 12:**
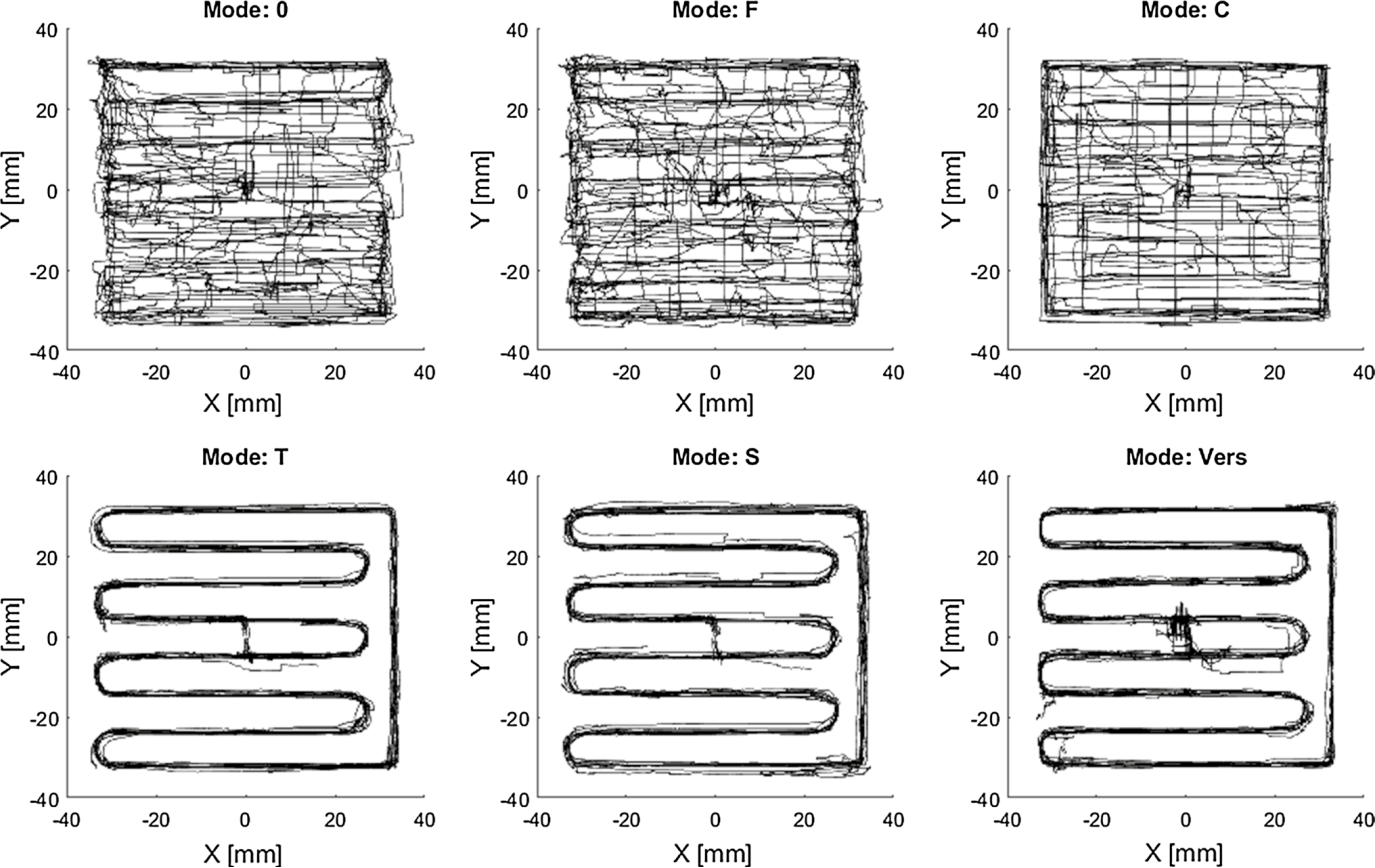
Exemplary superposition of all milling paths for P1 (partial knee replacement) (0 no haptic feedback, *F* haptic feedback, *C* constraint, *T* trajectory, *S* scaled, *Vers* versatile)

For assisted milling tasks (P1), statistically significant improvements with respect to effectiveness can be observed if motion is haptically constrained by boundaries (*C*). Furthermore, slight improvements by the remaining modes can also be observed but not statistically proven. Additionally, comparing modes *T* and *S* tendencies indicate a positive effect of scaling on task effectiveness. Efficiency is significantly improved for the two trajectory modes (*T*, *S*), when compared to no assistance (0) or solely force feedback (*F*) due to a more efficient path execution (Fig. 12), which is in accordance with the literature [12, 15].

When assistances cannot be offered in all DOF (P2) improvements with respect to the reference mode (0) cannot be observed, however, tendencies indicate a positive effect of scaling (comparison of *T* and *S*). Modes which solely provide a visual substitution of the force seem to achieve the best results with respect to effectiveness. This might be due to the rapidly decreasing force at the target depth and a resulting overshoot by the participants. Trajectory guidance seems to have a negative effect on the depth control (comparison of *F* and *T*, whose difference is guidance along a trajectory in the main visual plane). With respect to efficiency, similar trends as during P1 can be observed; however, no significant differences can be proven for P2.

Results regarding user satisfaction gathered for both phases combined indicate a significant reduction in perceived workload and an increased perceived usability for both trajectory modes (*T*, *S*). However, the perceived influence of participants on task execution is reduced and their perceived restriction increased for both phases for the trajectory modes. With the versatile mode (Vers), perceived influence and restriction are on a similar level as with other modes (0, *F*, *C*). Despite having the free choice, participants activated the trajectory guidance in 19 out of 20 cases (for both phases), which might be due to enhanced subjective user satisfaction. Furthermore, participants preferred the usage of haptic force feedback for P2 as 70% of participants decided to use it.

In conclusion, for tasks which can be assisted in three DOF (P1) trajectory guidance is able to improve efficiency and subjective user satisfaction, while boundary constraints are best with respect to effectiveness (i.e., target depth deviation). However, trajectory guidance might improve effectiveness as well according to the literature [12]. To achieve this, the stiffness in the depth direction, which was solely one-third of the stiffness of the boundary constraints, should be increased. If assistance cannot be offered in all DOF (P2), trajectories are rather disadvantageous and a visual substitution of the force feedback shows best results, though participants prefer to use haptic feedback. Findings in the literature also report on positive effects using additional auditory guidance and hypothesize that even multimodal applications including auditory, haptic and visual feedback are conceivable to further improve performance [36]. However, based on our results of PS1 an excessive amount of feedback modalities can also overburden users and should be considered.

Limitations of the study include that participants were informed about the currently active mode, which could have influenced their subjective workload ratings, and that participants were not surgeons. Furthermore, since assumptions of ANOVA could only be checked visually due to the limited number of participants, statistical results have to be treated with some reservation. Additionally, as the group of non-trajectory modes (0, *F*, *C*) was always presented before the remaining three modes (*T*, *S*, Vers) in the second main experiment learning effects may have influenced results. However, within each group learning effects constitute a random rather than a systematic error due to randomization for both experiments.

In conclusion, investigations show that advantages of *haptic assistance* and *haptic (senor) feedback* can be combined and if done correctly usability can be improved beyond exclusive use of one of the haptic information.
